# The MXL-3/SBP-1 Axis Is Responsible for Glucose-Dependent Fat Accumulation in *C. elegans*

**DOI:** 10.3390/genes8110307

**Published:** 2017-11-06

**Authors:** Fanny Mejia-Martinez, Berenice Franco-Juarez, Elizabeth Moreno-Arriola, Alain Hernández-Vázquez, Marco Martinez-Avila, Saul Gómez-Manzo, Jaime Marcial-Quino, Karla Carvajal, Antonio Velazquez-Arellano, Daniel Ortega-Cuellar

**Affiliations:** 1 Laboratorio de Nutrición Experimental, Instituto Nacional de Pediatría, Secretaría de Salud, Mexico City 04530, Mexico; fanny.mejia.510@gmail.com (F.M.-M.); mmartinezav@gmail.com (M.M.-A.); karla_ca@yahoo.com (K.C.); 2 Unidad de Genética de la Nutrición, Instituto de Investigaciones Biomédicas UNAM—Instituto Nacional de Pediatría, Mexico City 04530, Mexico; franco_beju@hotmail.com (B.F.-J.); elizamor86@hotmail.com (E.M.-A.); alain-hernandez@hotmail.com (A.H.-V.); velare@unam.mx (A.V.-A.); 3 Laboratorio de Bioquímica Genética, Instituto Nacional de Pediatría, Secretaría de Salud, Mexico City 04530, Mexico; saulmanzo@ciencias.unam.mx; 4 Consejo Nacional de Ciencia y Tecnología (CONACYT), Instituto Nacional de Pediatría, Secretaría de Salud, Mexico City 04530, Mexico; jmarcialqu@conacyt.mx

**Keywords:** *Caenorhabditis elegans*, glucose, fatty acid, energy metabolism, transcription factor, energy sensor

## Abstract

Chronic exposure to elevated glucose levels leads to fatty acid accumulation, which promotes the development of metabolic diseases such as obesity and type 2 diabetes. MXL-3 is a conserved transcriptional factor that modulates the inhibition of lipolysis in *Caenorhabditis elegans*. However, the role of MXL-3 in lipid metabolism during nutrient excess remains unknown. We hypothesized that inhibition of MXL-3 prevents glucose-dependent fat accumulation. Nematodes from wild-type N2, MXL-3::GFP and *sbp-1* or *mxl-3* null strains were grown on standard, high glucose or high glucose plus metformin plates for 24 h. Using laser-scanning confocal microscopy, we monitored the glucose-induced activation of MXL-3 labeled with GFP (MXL-3::GFP). Lipid levels were determined by Oil Red O (ORO) staining and gas chromatography/mass spectrometry, and gene expression was assessed by qRT-PCR. We found that high glucose activated MXL-3 by increasing its rate of nuclear entry, which in turn increased lipid levels via sterol regulatory element-binding protein (SBP-1). This activated critical genes that synthesize long chain unsaturated fatty acids (MUFAs and PUFAs) and repress lipolytic genes. Interestingly, the anti-diabetic drug metformin inhibited MXL-3 activation and subsequently prevented glucose-dependent fat accumulation. These findings highlight the importance of the MXL-3/SBP-1 axis in the regulation of lipid metabolism during nutritional excess and provide new insight into the mechanism by which metformin prevents lipid accumulation. This study also suggests that inhibition of MXL-3 may serve as a potential target for the treatment of chronic metabolic diseases, including obesity, type 2 diabetes, and cardiovascular disease.

## 1. Introduction

Hyperglycemia is a key risk factor for the development of diabetes mellitus and metabolic syndrome. Nowadays, both diseases have a substantial impact on public health and safety because they exacerbate the burden on health services [1,2]. These metabolic diseases result from an imbalance between the amounts of energy ingested and consumed and they are characterized by lipid dysregulation that affects energy homeostasis.

Even though several experimental and clinical attempts have been made to control both diabetes and metabolic syndrome, their prevalence continues to rise, contributing to the estimated parallel decline in worldwide health [3,4,5]. Given the complexity of the molecular mechanisms that underlie these diseases, a deep understanding of these mechanisms is essential to discover new treatments that prevent the development of metabolic diseases and improve quality of life. In particular, the discovery and modulation of regulatory elements, such as transcriptional factors, represents a strategy for combating pernicious effects of diabetes and other complications of the metabolic syndrome.

Although glucose is the main oxidative fuel source in eukaryotes, excessive glucose availability has detrimental effects (glucotoxicity) on organismal metabolism. Similar to its effect in humans, excess glucose exposure in *Caenorhabditis elegans* triggers toxic effects characterized by decreased lifespan, delayed reproduction and fertility, and excessive production of reactive oxygen species (ROS), in part due to mitochondrial dysfunction [6,7,8,9].

The transcriptional factor Myc-associated factor X (MAX) belongs to a family of transcription factors that contain basic helix-loop-helix motifs (b-HLH) and leucine zipper domains. MAX interacts with c-Myc (also a transcription factor member of the b-HLHzip family) to form a MAX–Myc heterocomplex that binds to DNA in specific E-boxes sequences (CACGTG); this interaction is indispensable for the regulation of gene expression [10,11]. Although little is known about the general mechanism that controls the MAX–Myc complex, MAX–Myc has the ability to activate or inhibit several genes that are involved in different cellular functions, including energy metabolism [12]. In fact, studies have shown that exposure of insulinoma cells to high glucose concentrations increases both c-Myc abundance and its association with its obligate heterodimer MAX to activate several glucose-responsive and de novo fatty acid synthesis genes, such as lactate dehydrogenase (LDH), L-pyruvate kinase (L-PK), pyruvate dehydrogenase (PDK-1), acetyl-CoA carboxylase (ACC), fatty acid synthetase (FASN) and stearoyl-CoA desaturase (SCD) [13]. Additionally, although the involvement of MAX has not been analyzed, endogenous levels of Myc promote de novo fatty acid synthesis [14,15], suggesting that the MAX/Myc network induces lipid accumulation via the incorporation of glucose-derived carbons into fatty acids.

Sterol regulatory element-binding protein (SREBP) is a major transcriptional regulator of lipogenic metabolism in mammals [16,17]. SREBP overexpression promotes its interaction with c-Myc to enhance the cellular reprograming of lipid metabolic genes [18]. However, whether the MAX/Myc/SREBP network is involved in MAX/Myc-mediated activation of the aforementioned genes is unknown.

*Caenorhabditis elegans*, a useful model to study energy balance and the regulation of lipid metabolism, has two MAX orthologous genes called MXL-1 and MXL-3 [19]. MXL-3 likely functions as a homodimer, unlike mammalian MAX; furthermore, MXL-3 does not appear to interact with other *C. elegans* Myc family members [20]. *C. elegans* MXL-3 modulates lipid metabolism by controlling specific lysosomal lipase *lipl-1* and *lipl-3* genes in response to nutrient availability [19]. Interestingly, global mapping and transcription factor activity analysis revealed that MXL-3 could positively regulate the *C. elegans* SREBP homolog SBP-1 [21]. Thus, it is plausible that MAX and MXL-3, in their respective species, play a critical role in regulating lipid metabolism, which contributes to the increased risk of developing obesity and related diseases.

In this study, we characterized a novel metabolic function of the MXL-3 transcription factor in the control of systemic lipid storage. A specific *mxl-3* knockout nematode exhibited decreased lipid content, even in the presence of excess lipogenic substrates, i.e., a high-glucose diet. Mechanistically, MXL-3 underwent nuclear translocation from the cytosol in response to a high-glucose diet, leading to the activation of a lipogenic program through the transcription of *sbp-1*, thereby modulating important lipogenic enzyme genes in an MXL-3-dependent manner. This effect was blunted by the drug metformin. Our data demonstrate that MXL-3 is an essential regulator of lipid metabolism and strongly suggest that MXL-3/SBP-1 may be a novel target for the development of therapeutic procedures that ameliorate or even prevent metabolic diseases.

## 2. Materials and Methods

### 2.1. Strains

All strains were obtained from the Caenorhabditis Genetics Center (CGC, University of Minnesota, Minneapolis, MN, USA) strain bank. The wild-type strain was *C. elegans* variant Bristol, N2 strain. The following mutants were used: RB1588 mxl-3 (ok1947) X, VC1886 *sbp-*1 (ok2363) III and UL1439 MXL-3::GFP unc-119 (ed3) III. Worms were cultured and handled according to standard laboratory protocols, as described previously [22]. Briefly, worms were grown on NGM-lite plates and maintained at 20 °C with *Escherichia coli* OP50-1 as the food source. When required, synchronized nematodes were maintained until the L4 larval state and transferred to new experimental NGM plates without glucose (control), with 100 mM glucose (glucose) or with 100 mM glucose plus 50 mM metformin (glucose/metformin). Glucose plates were prepared at a final concentration of 100 mM by adding glucose to the solid media and then allowing to spread for at least one day; metformin (Sigma-Aldrich, St. Louis, MO, USA) was added to melted media [23]. All experimental media included 49 μM 5-fluorodeoxyuridine (FUDR, Sigma-Aldrich). After 24 h, nematodes were collected and excess bacteria were quickly removed with M9 buffer. Unless otherwise mentioned, samples were frozen in liquid nitrogen and stored at −70 °C until analysis.

### 2.2. Fat Storage Staining

Fat storage was assayed as described previously using the Oil red O (Sigma-Aldrich) staining method with some modifications [24,25]. Worms were collected with M9 buffer, washed twice with 1X PBS buffer, and suspended in 100 μL of 1X PBS, to which an equal volume of 2X buffer (160 mM KCl, 40 mM NaCl, 14 mM Na_2_EGTA at pH 7.4) containing 2% paraformaldehyde was added. Worms were then washed three times with 1X PBS. After allowing the worms to settle, the buffer was aspirated, and 500 μL of freshly prepared ORO solution was added (0.5 g of ORO powder in 100 mL of 60% isopropanol; filter: 0.2 μm [26]). Animals were incubated overnight with rocking. The dye was removed, and the samples were washed with 200 μL of 1X PBS/0.01% Triton X-100. Worms were mounted on prepared agar slides. Images were acquired under differential interference contrast (DIC) and a rhodamine filter using a confocal FluoView FV1000 microscope (Olympus, Tokyo, Japan).

### 2.3. Fluorescence Imaging

Fluorescence imaging was performed as described previously, with some modifications [27]. Briefly, MXL-3::GFP (UL1439) transgenic worms were harvested and washed three times with cold M9 buffer, then once with 1X PBS/0.05% TritonX-100, and fixed with buffer (50% methanol, 20 mM EGTA, 1X PBS solution) containing 4% paraformaldehyde. Cells were permeabilized by performing two freeze–thaw cycles (liquid nitrogen/60 °C water bath) followed by gentle rocking for 24 h at 4 °C. After this procedure, worms were washed with 0.05% TritonX-100 in 1X PBS and incubated in 0.3% H_2_O_2_ in 20 mM sodium borate (pH 9.6) for 15 min. Finally, samples were washed with 1X PBS. Nuclei were visualized via DAPI (4,6-diamidino-2-phenylindole) staining. Fluorescence was examined at 358 nm using a confocal FluoView FV1000 microscope (Olympus).

### 2.4. RNA Extraction and Quantitative RT-PCR

Prior to total mRNA quantification, worms were lysed by adding 100 μL of lysis buffer (5 mM Tris pH 8.0, 0.5% Triton X-100, 0.5% Tween 20, 0.25 mM EDTA and 2 mg/mL proteinase K) and incubating at 65 °C for 10 min. To inactivate the proteinase K, samples were heated to 85 °C for 1 min and immediately cooled on ice [28]. Total RNA was extracted by adding 300 μL of Trizol to 100 μL of worm lysate following the manufacturer’s instructions. cDNA was obtained using random hexamer oligonucleotides and M-MLV reverse transcriptase (Invitrogen, Carlsbad, CA, USA). Expression analyses were performed using Taqman (Applied Biosystems, Foster City, CA, USA) probes, which were detected using the StepOne (Thermo Fisher Scientific, Inc., Waltham, MA, USA) System and StepOne Software v2.2 (ABI instruments, Carlsbad, CA, USA). mRNA levels were normalized to eukaryotic 18S rRNA gene expression as an endogenous control.

### 2.5. Lipid Extraction and Gas Chromatography/Mass Spectrometry Assays

Fatty acid composition was determined by producing fatty-acid methyl esters (FAMEs). Total fatty acids were extracted via the chloroform-methanol (2:1, *v*/*v*) method and incubated at 95 °C for 1 h in 0.2 mL of toluene, 1.76 mL of methanol and 0.04 mL of H_2_SO_4_ [29,30]. The concentration and composition of FAMEs were evaluated, as described previously [31]. Briefly, Gas Chromatography/Mass Spectrometry (GC/MS) was performed using an Agilent 6890N gas chromatograph (Agilent Technologies, Santa Clara, CA, USA) equipped with an HP-5MS column (30 m × 0.25 mm I.D. × 0.25 μm, Agilent) containing ultrapure helium as a carrier gas, and 1 μL of each sample was injected at a 10:1 split ratio. The initial oven temperature was set to 150 °C, increased to 310 °C at 8 °C per min, and held for 15 min at this temperature. Mass detection was performed as previously described for a JMS-GC System [31]. Fatty acid abundance was expressed as the percent of total fatty acids in the N2 control strain for each sample.

## 3. Results

### 3.1. MXL-3 Is Involved in Lipid Accumulation Due to High Glucose Exposure in *C. elegans*

**Figure 1:**
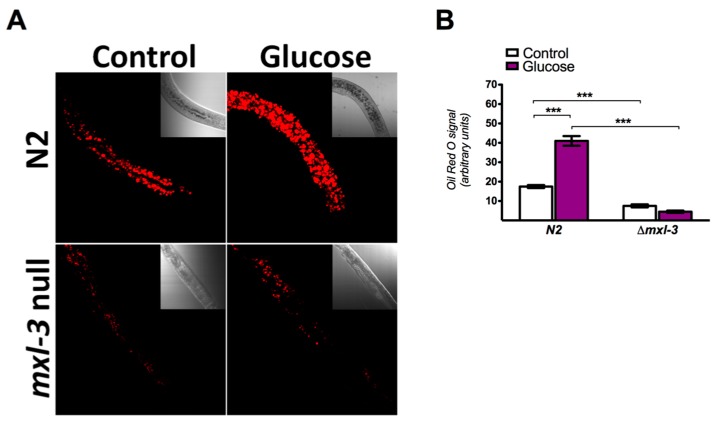
MXL-3 is required for high glucose-induced lipid accumulation in *C. elegans*. Oil Red O staining of total lipids in fixed worms grown under control or high glucose conditions. (**A**) Representative image of wild-type (N2) and *mxl-3* mutant (*ok1947 null*) nematode strains showing stained intracellular lipid accumulation during exposure to high glucose (red). (**B**) Histogram of the quantification of ORO-stained lipid droplets shown in A. Data represent the mean ± SEM of three independent experiments; *** *p* < 0.001, one-way ANOVA with Bonferroni’s post hoc test using GraphPad Prism.

To investigate whether the MXL-3 transcription factor facilitates the excess lipid accumulation induced by high glucose exposure in *C. elegans*, semi-quantitative analysis of neutral lipids stained with ORO was performed in fixed wild-type (N2) and *mxl-3* null mutant (*mxl-3/ok1947*) animals. Consistent with previous reports [7], we found that high glucose exposure for 24 h significantly induced lipid accumulation in N2 animals, as demonstrated by an increase in the intensity of ORO staining (Figure 1A,B). Next, we determined whether lipid accumulation due to a high-glucose diet is influenced by MXL-3. Ablation of *mxl-3* substantially reduced ORO staining compared with that observed for the N2 control strain (Figure 1A,B), whereas the augmented lipid storage observed in N2 under high glucose was blunted in MXL-3-null animals, even when the mutant was grown under the same conditions, suggesting that fat accumulation is mediated by the MXL-3 transcription factor.

### 3.2. MXL-3 Actively Shuttles between the Cytoplasm and Nucleus in Response to High Glucose

**Figure 2:**
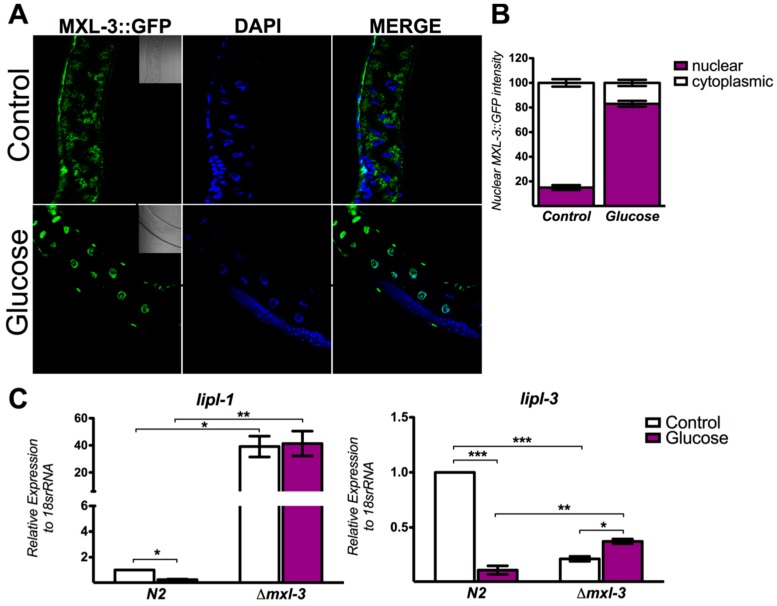
Glucose activates MXL-3 by increasing its rate of nuclear entry. High glucose promotes MXL-3 nuclear entry and represses *lipl-1* and *lipl-3* expression. (**A**) Representative confocal fluorescent images that show cytoplasmic MXL-3::GFP (in control conditions) and its nuclear localization during high glucose exposure (green dots). Nuclei are identified by DAPI staining (blue). (**B**) Nuclear MXL-3::GFP intensity by glucose relative to nematodes fed with control diet. (**C**) mRNA quantification of *lipl-*1 and *lipl-3* was performed using qRT-PCR in the N2 and *mxl-3* mutant (*ok1947 null*) nematode strains. The relative expression of each gene was normalized to endogenous 18S rRNA gene expression. Data represent the mean ± SEM of three independent experiments; * *p* < 0.05, ** *p* < 0.01, *** *p* < 0.001, one-way ANOVA with Bonferroni’s post hoc test using GraphPad Prism.

MXL-3 actively shuttles between the cytoplasm and nucleus in response to nutrient availability [19]. Therefore, it seems plausible that glucose might affect the cellular localization of MXL-3. To determine whether high glucose exposure modifies the nucleocytoplasmic shuttling of MXL-3::GFP, transgenic worms were grown on media containing high glucose (100 mM) or no glucose (control) for 24 h. Glucose exposure resulted in the major accumulation of MXL-3::GFP in the nucleus compared to that in the controls, suggesting that glucose augments its regulatory activity (Figure 2A,B). To verify if nuclear localization correlates with transcriptional activity, we measured several known target genes of MXL-3 by performing quantitative RT-PCR (qRT-PCR). The expression levels of the specific lysosomal lipases *lipl-1* and *lipl-3* were markedly downregulated after N2 nematodes were fed high glucose for 24 h compared to the levels in worms grown under control conditions; this effect was abolished in *mxl-3* null mutants (Figure 2C). Thus, MXL-3 nuclear localization induced by glucose enhances the transcriptional repression of these genes.

### 3.3. MXL-3 Regulates Lipid Synthesis Through SBP-1

**Figure 3:**
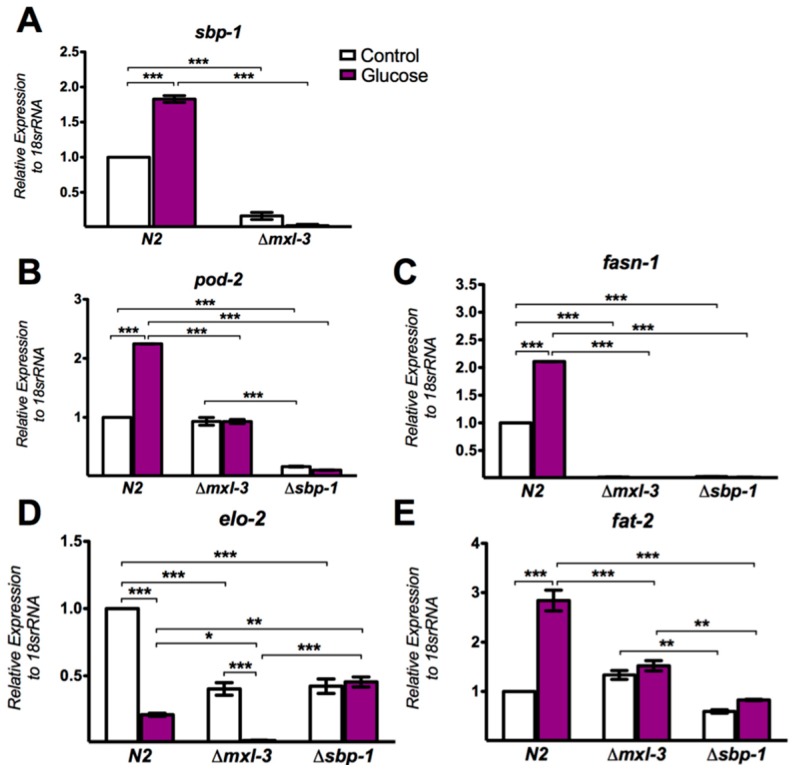
Transcriptional control of lipid-related genes by MXL-3. The relative mRNA expression levels of several genes: (**A**) *sbp-1*; (**B**) *pod-2*; (**C**) *fasn-1*; (**D**) *elo-2*; and (**E**) *fat-2* in N2 wild-type or *mxl-3* and *sbp-1* mutant strains. Mutation of *mxl-3* significantly decreased *sbp-1* mRNA levels under both control and high-glucose conditions. Similar results were observed for *elo-2* and *fasn-1*, whereas no effects on *pod-2* and *fat-2* were observed. The relative expression of each gene was normalized to endogenous 18S rRNA gene expression. Data represent the mean ± SEM of three independent experiments; * *p* < 0.05, ** *p* < 0.01, *** *p* < 0.001, one-way ANOVA with Bonferroni’s post hoc test using GraphPad Prism.

As observed in mammals, the transcription factor SREBP ortholog in *C. elegans*, SBP-1, induces lipid synthesis by activating lipogenic genes such as acetyl-CoA carboxylase (*pod-2*), fatty acid synthase (*fasn-1*), elongase (*elo-2*) and ∆12 desaturase (*fat-2*) [32]. A previous study employing an assay of transcriptional activity proposed that MXL-3 positively regulates SBP-1 expression [21], suggesting a new physiological role for MXL-3 in the lipid biosynthesis signal transduction pathway. To determine if SBP-1 induces lipid accumulation in an MXL-3-dependent manner under high glucose conditions, we first measured the mRNA expression levels of sbp-1 during glucose excess. As expected, the expression of sbp-1 mRNA was significantly higher in nematodes treated with high glucose (Figure 3A), suggesting augmented activity of this transcription factor. To establish MXL-3- and SBP-1-dependent gene regulation, we grew *mxl-*3 null mutants (ok1947 strain) on high glucose and measured the mRNA levels of both *sbp-1* and some of their known target genes. MXL-3 was necessary for full *sbp-1* expression, as the *mxl-3* (ok1947) null mutant failed to properly activate *sbp-1* gene expression (Figure 3A). Similarly, genes that were highly expressed under glucose, specifically *pod-2*, *fasn-1*, and *fat-2*, were blunted by the absence of MXL-3 (Figure 3B–D), indicating that this factor is an additional element required for the appropriate expression of SBP-1-dependent genes. *elo-2* expression was decreased under high glucose, even in the N2 and *mxl-3* null mutant strains. To determine to what extent MXL-3 contributes to glucose-regulated gene expression through SBP-1, similar studies were performed in *sbp-1* mutants. As expected, the expression levels of *pod-2*, *fasn-1*, *elo-2* and *fat-2* were not induced in response to a high-glucose diet (Figure 3). Taken together, our data suggest that MXL-3 might be important for promoting a selective lipogenic program that is dependent on a high-glucose diet through SBP-1.

### 3.4. MXL-3 Is Required to Maintain Fat Stores and De Novo Fat Synthesis

**Figure 4:**
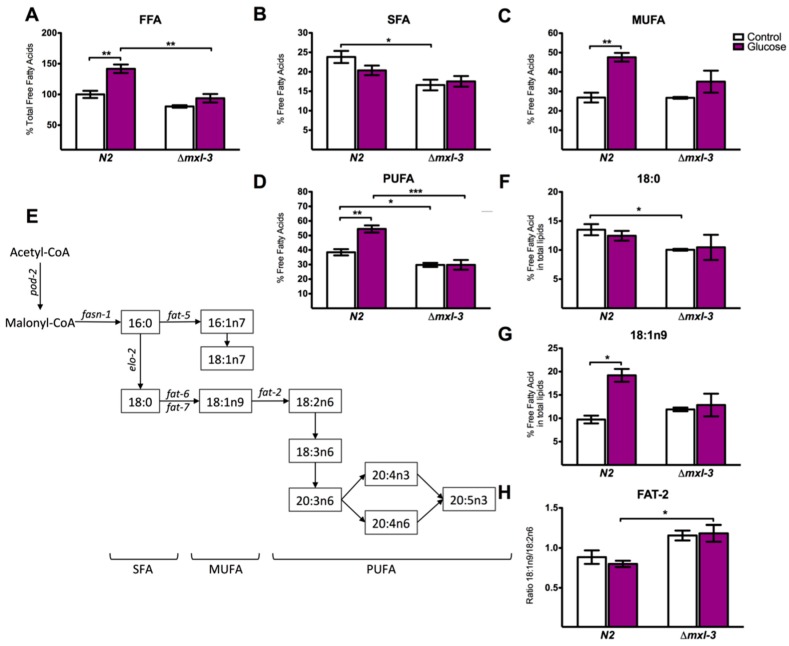
MXL-3 participates in the regulation of fatty acid synthesis, elongation and desaturation. Fat content changed markedly in worms exposed to glucose. (**A**–**D**) N2 worms administered glucose demonstrated augmented FFAs, MUFAs, and PUFAs, whereas these FAs were decreased in the *mxl-3* mutant, even under high-glucose conditions. In contrast, SFA decreased under the same conditions. (**E**) Schematic representation of the SFA, MUFA, and PUFA synthesis pathways. (**F**) 18:0 (stearic acid) was decreased and (**G**) 18:1n9 (oleic acid) was augmented. These responses were absent in the *mxl-3* mutant strain. (**H**) FAT-2 activity was indirectly measured from substrate/product ratios. If the relationship increased, a decrease in activity was inferred. Each graph shows the mean ± SEM of three independent experiments; * *p* < 0.05, ** *p* < 0.01, *** *p* < 0.001, one-way ANOVA with Bonferroni’s post hoc test using GraphPad Prism.

**Figure A1:**
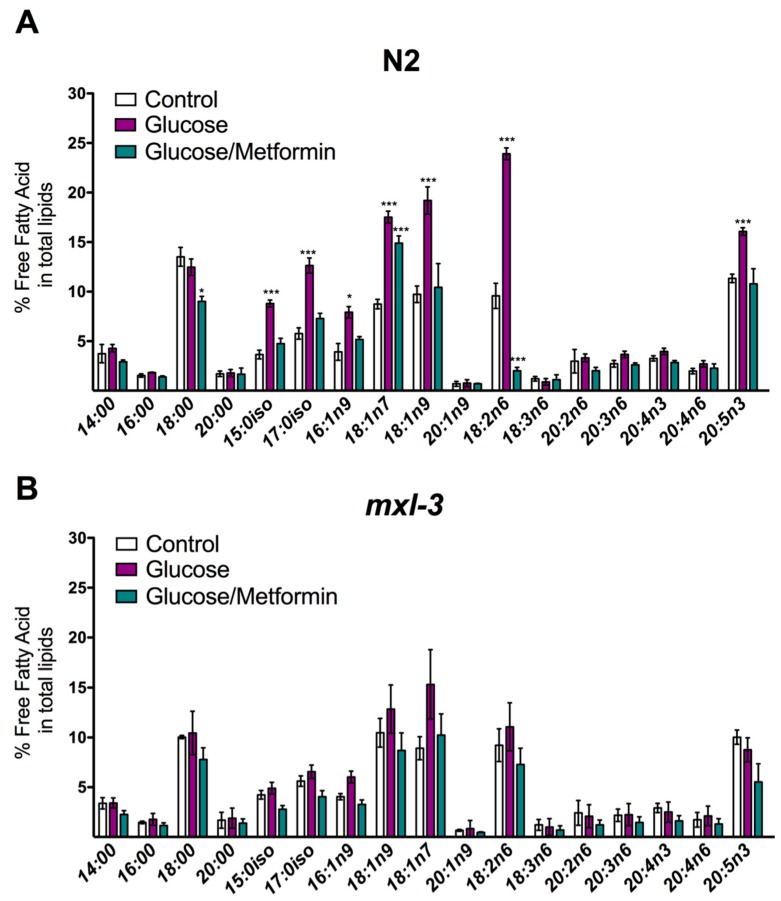
The striking lipid profile shift after glucose and metformin exposure is controlled by MXL-3. A subset of 17 fatty acids is shown in the N2 and *mxl-3* mutant strains treated with high glucose and high glucose plus metformin. (**A**) FFAs that increased due to glucose exposure showed a clear tendency to return to the levels of the control group in the presence of metformin. (**B**) FFAs are unable to change in the *mxl-3* mutant due to glucose or metformin. Graphs depict the mean ± SEM of three independent experiments; * *p* < 0.05, *** *p* < 0.001 vs. fatty acids in the control group, one-way ANOVA with Bonferroni’s post hoc test using GraphPad Prism.

**Figure A2:**
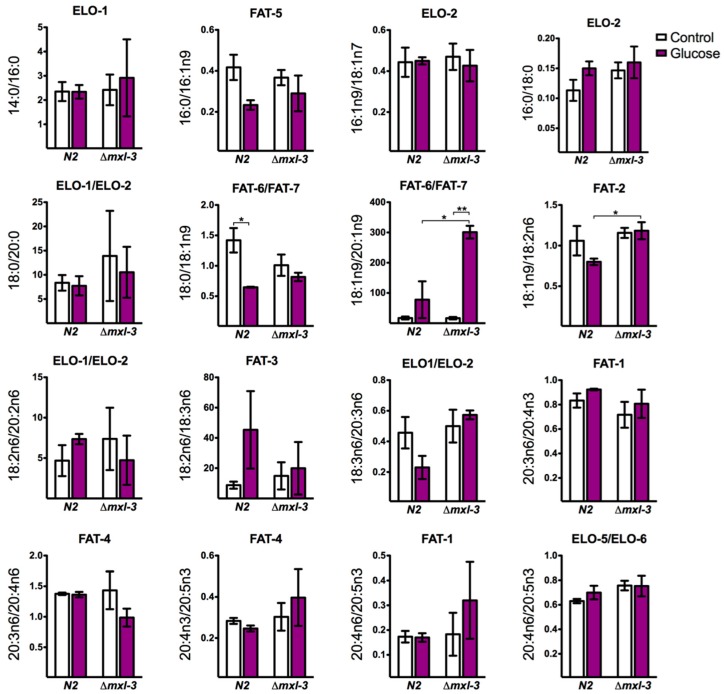
Indirect measurement of the fatty acid biosynthetic pathway. Specific steps in the fatty acid biosynthetic pathway were indirectly measured from substrate/product ratios. If the relationship increased, a decrease in activity was inferred. Each graph shows the mean ± SEM of three independent experiments; * *p* < 0.05, ** *p* < 0.01 vs. fatty acids in the control group, one-way ANOVA with Bonferroni’s post hoc test using GraphPad Prism.

Since MXL-3 promotes transcriptional changes under a high-glucose diet through SBP-1, we determined whether the observed changes were also reflected in fat and lipid metabolism. Using GC/MS, we measured the composition of free fatty acids (FFA) in the *mxl-3* mutant strain. Consistent with ORO staining (Figure 1A), worms lacking MXL-3 showed decreased FFA content (Figure 4A and Figure A1). We also analyzed the three FFA lipid classes, saturated fatty acids (SFAs), mono-unsaturated fatty acids (MUFAs), and poly-unsaturated fatty acids (PUFAs), and found that MXL-3 triggers a metabolic shift that decreases SFAs and PUFAs (Figure 4B,D); MUFAs were not modified by the absence of MXL-3 (Figure 4C). In *mxl-3* mutants, we observed a decrease in stearic acid (18:0), a fatty acid obtained by elongation that is dependent on ELO-2, an SBP-1-dependent elongase (Figure 4F). Additionally, in the absence of MXL-3, the 18:1n9/18:2n6 (oleic acid/linoleic acid) ratio was decreased (Figure 4G and Figure A2), while oleic acid was increased (Figure 4H), suggesting FAT-2 (Δ12) desaturase blockage controls the conversion of fatty acids to PUFAs (Figure 4E). When the *mxl-3* mutant was subjected to a high-glucose diet and its lipid profile was analyzed by GC/MS, we did not observe FFA accumulation or SFA/PUFA changes, which were normally observed in the wild-type strain. MUFA content was slightly increased by glucose (Figure 4A–D and Figure A1). Together, these data suggest that MXL-3 is as necessary as SBP-1 for the adequate synthesis and elongation of fatty acids, and MXL-3 is involved in the same steps mediated by SBP-1. These findings reinforce the hypothesis that MXL-3 acts through SBP-1 to regulate lipid metabolism.

### 3.5. Metformin Decreases Lipid Overload through MXL-3

**Figure 5:**
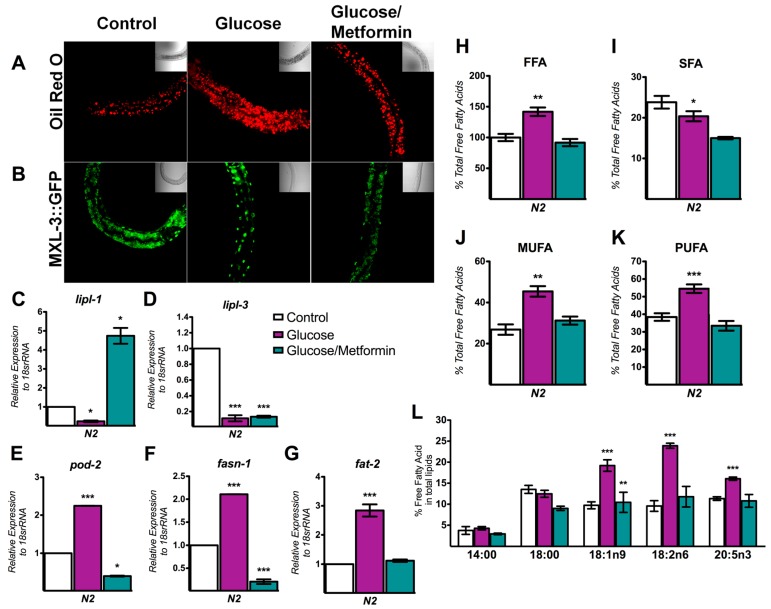
Metformin protects *C. elegans* against glucose-induced fat accumulation by MXL-3. Metformin decreased lipid storage and modified FFA profiles through MXL-3. (**A**,**B**) Representative images of lipid droplets indicated by ORO staining (red dots) or the subcellular localization of MXL-3::GFP (green dots) under control, glucose and glucose/metformin conditions. (**C**–**G**) mRNA gene expression analysis was performed by performing qRT-PCR on wild-type N2 under the same experimental conditions: (**C**) *lipl-1*; (**D**) *lipl-3*; (**E**) *fasn-1*; (**F**) *pod-2*; and (**G**) *fat-2*. The relative expression of each gene was normalized to endogenous 18S rRNA gene expression. (**H**–**L**) Total FFAs and their subgroups are modified similar to the control group when metformin us added: (**H**) FFA; (**I**) SFA; (**J**) MUFA; (**K**) PUFA; and (**L**) fatty acid profile. Data represent the mean ± SEM of three independent experiments; * *p* < 0.05, ** *p* < 0.01, *** *p* < 0.001, one-way ANOVA with Bonferroni’s post hoc test using GraphPad Prism.

Metformin, a drug that is broadly prescribed to treat type-2 diabetes and obesity, is beneficial because it modulates several transcriptional elements that affect lipid storage [33]. To evaluate the effects of metformin on the glucose-dependent accumulation of lipid droplets, the wild-type N2 strain was grown with high glucose and simultaneously treated with metformin (glucose-metformin). Glucose-dependent lipid droplet accumulation was diminished in response to metformin, as evidenced by the reduced fluorescence of ORO staining close to control levels (Figure 5A). Since we showed that glucose availability modulated the cellular location of MXL-3, we next evaluated the subcellular distribution of MXL-3 upon treatment with metformin (24 h). Interestingly, metformin-treated worms grown in glucose-containing media showed a diminishment of nuclear content of MXL-3::GFP from their nuclei, resulting in cytoplasmic accumulation similar to untreated worms (Figure 5B). Thus, the localization of MXL-3 in the cytoplasm upon metformin treatment in the presence of glucose may be one mechanism underlying reduced lipid storage. Consistent with the exit of MXL-3 from the nucleus, expression of the MXL-3 target gene *lipl-1* increased to the same extent, while *lipl-3* expression did not recover (Figure 5C,D). Similarly, when MXL-3 was inhibited by metformin, the MXL-3-dependent expression of SBP-1 target genes, including *fasn-1*, *pod-2*, and *fat-2*, returned to control levels in N2 nematodes (Figure 5E–G). Furthermore, based on MXL-3 cytoplasmic localization, when wild-type worms were grown in the presence of both glucose and metformin and then lipid-related gene expression and lipid stores were assessed, FFAs were decreased by metformin versus the glucose group and mimicked the lipid levels observed under control conditions, even in the presence of high glucose (Figure 5H and Figure A1). This effect was more evident for MUFAs and PUFAs, which returned to control levels (Figure 5I–K). Specifically, the fatty acid moieties that had a clear tendency to decrease to control levels under glucose-metformin conditions were the myristic, stearic, oleic, linoleic and eicosapentaenoic acids (14:0, 18:0, 18:1n9, 18:2n6, and 20:5n3, respectively, Figure 5L). This lipid profile was surprising because it was similar to the *mxl-3 null* mutant profile (Figure A1). Based on these results, metformin decreased lipid storage and modified the FFA profile via an MXL-3- and SBP-1-dependent mechanism.

## 4. Discussion

In this study, we determined that MXL-3 is activated by a high-glucose diet and, together with SBP-1, promotes fat accumulation. This effect was mediated by the increased expression of genes that mediate fatty acid synthesis and desaturation. Furthermore, lipid accumulation under glucose-rich conditions was also dependent on MXL-3-dependent lipolysis. Moreover, the anti-diabetic drug metformin prevented MXL-3 activation, suggesting a new key role for MXL-3 in the mechanism underlying metformin action on lipid accumulation. Thus, MXL-3 is a transcription factor that acts at the nexus of metabolic pathways to stimulate fat accumulation.

### 4.1. MXL-3 Senses Nutrient Availability and Regulates the Obese Phenotype during High Glucose Exposure

The key finding of our study was the identification of MXL-3 as a primary activator of an obese phenotype that has previously been shown by others to be derived from lipid accumulation via high glucose ingestion [6,7,8,9]. Myc-associated factor X contains a basic helix–loop–helix motif (b-HLH) and a leucine zipper domain and functions at the core of a transcription factor network that controls diverse members of the Myc family. Indeed, the heterodimer MAX-Myc binds to E-box sequences (CACGTG) to activate or repress the transcription of several genes, including those for glycolysis [10,11]. Importantly, in several processes such as the cell cycle and apoptosis, there is a strict requirement for MAX to ensure adequate Myc function. However, in *Drosophila* spp., a Myc mutant lacking a Max-interaction domain retained partial activity [34]. *C. elegans* contains two MAX orthologs, MXL-1 and MXL-3, the latter of which regulates lipolysis in response to nutrient levels [19]. However, the role of MXL-3 in the development of metabolic diseases, such as obesity dependent on a high-glucose diet, remains poorly studied. Therefore, the importance of MXL-3 activity in the control of lipid accumulation under high glucose exposure was evaluated in this study.

According to O’Rourke and Ruvkun, MXL-3 moves to the cytoplasm during nutrient deprivation and de-represses lysosomal lipolysis (lipophagy). This is a critical mechanism by which most cells catabolize lipids as an energy source [19]. Interestingly, we observed that MXL-3::GFP shuttled from the cytoplasm to the nucleus under high-glucose conditions, suggesting MXL-3 is a sensor of intracellular glucose metabolism; indeed, during nutrient deprivation, MXL-3 was expelled from the nucleus, whereas under high-glucose conditions, MXL-3 occupies the promoters of its targets to modulate gene expression. Consistent with the glucose-dependent occupancy of MXL-3 in the nuclei, the expression levels of *lipl-1* and *lipl-3* were diminished by glucose excess, resulting in repression of the lipophagic process. These findings suggest that MXL-3 activation is responsible for lipid accumulation.

### 4.2. MXL-3 Converges in the Complex Lipid Biosynthesis Signaling Pathway

Energy homeostasis is maintained by an extremely complex network of signaling pathways that operate to mediate different aspects of the fatty acid balance to maintain energetic resources. SBP-1, known as SREBP1c in mammals, acts as a critical lipogenic transcriptional factor that promotes lipid synthesis by inducing genes such as *pod-2*, *fasn-1* and (*elo-2* [32]. However, less is known about its upstream regulation. Recently, employing a transcriptional activity assay, MacNeil et al. proposed that MXL-3 positively regulates *sbp-1* expression [21]. Since MXL-3 increased lipid storage and given the key role of SBP-1 in lipid metabolism, we tested the possible relationship between both transcription factors. Interestingly, in animals that lacked *mxl-3*, we showed diminished *sbp-1* gene expression. The effects of glucose observed in wild-type strains and the near doubling of *sbp-1* expression in the N2 strain with glucose were avoided in *mxl-3* mutants. Similarly, we observed a dependence on MXL-3 for the adequate expression of SBP-1-dependent lipogenic genes, including *pod-2*, *fasn-1* and *fat-2*, when glucose was administered. Based on these findings, transcriptional lipogenic processes are not only regulated by SBP-1 but also by MXL-3. Thus, we propose a new critical physiological role for MXL-3 in the complex lipid biosynthesis signal transduction pathway.

Fat regulation in living organisms, including *C. elegans*, involves a complex network of genes and signaling pathways. Here, we showed that MXL-3 is in part responsible for the observed transcriptional changes that result in lipid accumulation during a high-glucose diet. Our analysis of fatty acid composition using GC/MS showed that the absence of *mxl-3* decreases total FFAs in a similar fashion as previously reported in an *sbp-1 RNAi* strain [9]. When we analyzed FFAs grouped in families according to their saturation levels, we showed that the absence of the MXL-3 transcription factor decreased SFAs and PUFAs but did not change MUFAs. These results might be explained by blockage of the FAT-2 (∆12) desaturase, an enzyme that catalyzes a critical SBP-1-dependent step, because there was a decrease in calculated activity leading to a decrease in the conversion of fatty acids to PUFAs. This led to decreased de novo synthesis from acetyl-coA because the genes that carry out this process were also reduced.

For de novo synthesis, two multifunctional enzymes, acetyl-CoA carboxylase (POD-2) and fatty acid synthase (FASN-1), both of which are SBP-1 targets, are necessary to synthesize palmitic acid (16:0) [32,35]. Palmitic acid is integrated into TAGs or is modified by fatty acid elongases and desaturases (*elo* and *fat* genes, respectively) to form a variety of long-chain UFAs [35]. In *mxl-3* mutants, we observed a decrease in stearic acid (18:0), a fatty acid obtained by elongation dependent on ELO-2, a palmitic acid elongase that is itself dependent on SBP-1 [32]. In addition, the ratio of 18:1n9/18:2n6 (oleic acid/linoleic acid) was decreased while oleic acid increased, confirming the decreased synthesis of PUFAs in *mxl-3* mutants; similar levels of PUFAs were observed in an *sbp-1* RNAi strain [9]. These changes in fatty acid composition may affect energy storage functions as well as membrane properties associated with signal transduction mechanisms.

Thus, the key finding of our study is the identification of MXL-3, which acts in concert with SBP-1, as an essential component for fatty acid metabolism. Interestingly, the overlapping functions of these two transcription factors might be explained by the fact that both are helix–loop–helix proteins with canonical E-box binding activities [36], thus suggesting a possible interplay between these transcription factors to regulate lipid metabolism.

### 4.3. MXL-3 Is a New Player That Mediates the Protective Effect of Metformin against Lipid Accumulation

Metformin is one of the most commonly prescribed drugs for type II diabetes and obesity [33]. Previously, metformin was found to decrease lipid concentrations in *C. elegans* [23]. Therefore, we speculated that metformin might be responsible for regulating the biological function of MXL-3. We demonstrated that metformin enhance cytoplasmic localization of MXL-3, even in glucose-treated worms. These results are consistent with the fact that metformin triggers caloric restriction-like effects in *C. elegans* [37], promoting changes in MXL-3 cellular localization that have been observed during dietary restriction [19]. Similar effects on nucleocytoplasmic shuttling have also been described with other transcription factors, such as FOXO1 (Forkhead box protein O1) and CRTC2 (CREB Regulated Transcription Coactivator 2) [38,39,40]. Thus, our studies strongly suggest that regulation of MXL-3 pathway crosstalk with SBP-1 by metformin plays an important role in ameliorating lipid accumulation, a mechanism that was unknown until this work.

MXL-3 might modulate the cellular localization of DAF-16/FOX0, a pro-longevity and lipid-mobilizing transcription factor of the FOXO-family that is cytoplasmic under physiological conditions, because loss of MXL-3 function induces its nuclear localization, suggesting a relationship between both transcription factors [41]. Interestingly, a recent study suggested a positive feedback loop involving DAF-16 and AMPK, the latter of which is an evolutionarily conserved sensor of cellular energy status under low energy levels or metformin [42,43,44]. Consistent with this, we speculate that metformin, via AMPK induction, may inhibit MXL-3 to activate DAF-16 and trigger the activation of genes required for β-oxidation and desaturation, such as *nhr-49*, to diminish systemic lipid accumulation. These possibilities warrant further exploration.

## 5. Conclusions

In summary, our data showed that the increased expression of the transcription factor MXL-3 is associated with an increased activity of SBP-1, suggesting that this couple mediates the upstream regulation of lipid metabolism in nematodes exposed to a high-glucose diet. These observations imply that increased MXL-3 activity in the organism is an important factor in the development of pathogenesis of lipid accumulation. Therefore, we reasonably infer that MXL-3 antagonist (such as metformin) may serve a suitable mechanism for the pharmacology.
